# The Influence of Energy Levels and Defects on the Thermoluminescence of LiF: SiO_5_ Phosphors Doped with Ce^3+^

**DOI:** 10.3390/ijms26073183

**Published:** 2025-03-29

**Authors:** Habtamu F. Etefa, Xolani G. Mbuyise, Fikadu T. Geldasa, Genene T. Mola, Makaiko L. Chithambo, Francis B. Dejene

**Affiliations:** 1 Department of Physics, Faculty of Natural Sciences, Walter Sisulu University, Mthatha Campus, Nelson Mandela Drive Private Bag X1, Mthatha 5117, South Africa; fikadutakele2008@gmail.com (F.T.G.); fdejene@wsu.ac.za (F.B.D.); 2 School of Education, University of KwaZulu-Natal, Edgewood Campus, Admin Building LG Floor, Room 140, Durban 4000, South Africa; 3 School of Chemistry & Physics, University of KwaZulu-Natal, Pietermaritzburg Campus, Private Bag X01, Scottsville 3209, South Africa; 4 Department of Physics & Electronics, Rhodes University, Artillery Road, P.O. Box 94, Grahamstown 6139, South Africa

**Keywords:** TL luminescence, phosphor, dose, trap depth, phosphorescence

## Abstract

The morphological, structural, and thermoluminescence (TL) properties of LiF:SiO_5_ doped with Ce^3+^ solid powder phosphor were systematically analyzed. X-ray diffraction (XRD) confirmed the crystalline nature of single-phase LiF:SiO_5_:Ce^3+^ nanoparticles (NPs), with crystalline size (D) determined using the Williamson–Hall (W–H) and Scherrer methods. Ce^3+^ doping induced structural modifications, reflected in variations of full width at half maximum (FWHM), strain, and stress values. The TL glow curve revealed two distinct peaks at approximately 64 °C and 134 °C, shedding light on the electron capture and release mechanisms following beta irradiation. A dose-dependent study demonstrated that TL intensity increased proportionally with radiation exposure, showing a superlinearity relationship up to 6 Gy. Additionally, investigations into different heating rates indicated only a slight shift in peak of the temperature, confirming the thermal stability of the materials. This study provides valuable insights into the TL behavior of LiF:SiO_5_:Ce^3+^, making it a promising candidate for radiation dosimetry and luminescence applications.

## 1. Introduction

The term “thermoluminescence” originates from the idea that heat is the primary energy source for luminescence. However, this is inaccurate, as it contrasts with a substance’s radiation when heated to a bright light. For a substance to exhibit thermoluminescence, it must first have been exposed to radiation. While heating serves only as a catalyst for releasing the absorbed energy, that energy is derived from the initial radiation. Thermoluminescence is merely one of many luminescence phenomena [1,2]. Historically, the term “phosphorescence” has been used to describe a range of phenomena characterized by afterglows. This terminology distinguishes between long-lived emissions and the rapid emissions observed in fluorescence, which occur over a brief time frame (less than 108 s^−1^) [3,4]. Until recently, “phosphorescence” was used phenomenologically to differentiate between fluorescence and afterglow duration [5]. This has led to ambiguity, as the term describes the luminescence of organic and inorganic substances despite their different mechanistic origins. Factors such as temperature, oxygen availability, and molecular aggregation significantly influence the decay of phosphorescent light.

While it is super desirable to have metal-free organic dyes that show persistent phosphorescence in everyday conditions, organic materials are often the go-to choice because they are cheaper, biocompatible, easy to handle, and come with a variety of substitutes while still being relatively stable. Thus, the phosphorescence quenching due to vibrational relaxation during molecular collisions usually leads to lower quantum yields for these emissions in organic dyes at room temperature. Since 1944, there has been a push to improve the theory and practice of phosphorescence because “persistent luminescence” could really impact our lives. In 2007, a cool new imaging technique used phosphorescent rare-earth-doped nanoparticles with a high signal-to-noise ratio and long-lasting light emission in the red to near-infrared range. These cutting-edge optical labels are the newest step in bioimaging technology, allowing for deeper tissue penetration [6,7]. Transitional metals and rare earth ions are used in phosphors for different display applications. A lot of research is focusing on boosting the luminous efficiency of rare-earth-doped phosphor materials to optimize their properties for things like display panels and field emission display detectors [8,9,10]. This improvement comes from their impressive brightness, chemical stability, and wide industrial use.

Phosphor materials with efficient thermoluminescence (TL) properties have gained significant attention in radiation dosimetry, optoelectronics, and luminescence-based applications [11,12,13]. Among them, lithium fluoride (LiF)-based phosphors have been extensively studied due to their high radiation sensitivity, stability, and tunable optical properties [14]. Incorporating rare-earth dopants, such as Ce^3+^, into LiF matrices further enhances their luminescence efficiency and electronic trapping characteristics, making them ideal for dosimetric applications [15]. The TL response of phosphors is largely influenced by their structural properties, including crystallinity, defect states, and dopant-induced modifications [16,17]. LiF:SiO_5_, a promising phosphor material, exhibits enhanced charge-trapping mechanisms when doped with Ce^3+^ ions [18,19]. These dopants introduce localized energy states within the bandgap, influencing charge carrier dynamics and improving TL performance. Understanding these structural and electronic modifications is crucial for optimizing the material’s dosimetric response and ensuring its reliability in radiation detection technologies.

This article presents a complementary approach for localizing impurity energy levels in LiF:SiO_5_, which is doped with Ce^3+^ ions, utilizing the thermoluminescence phenomenon. LiF is typically sensitized by materials that emit broadband light. The combination of SiO5 transition metal ions and rare earth ions doped with Ce^3+^ results in visible luminescence; thus, we selected these materials due to their compatibility with the absorption spectra of LiF phosphor materials. Radiance can be stimulated as a dopant in LiF:SiO_5_ lattices; this emission is fundamentally a property of the Ce^3+^ rare earth ion, rather than the host material itself [20].

This study offers a comprehensive examination of Ce^3+^-doped LiF:SiO_5_ phosphors, distinguishing itself from previous research through enhanced thermal stability, precise TL glow curve analysis, and an innovative correlation between structural and dosimetric properties. The higher activation energy compared to other Ce^3+^-based phosphors, alongside its strong mechanical and optical stability, makes LiF:SiO_5_:Ce^3+^ a promising candidate for radiation dosimetry and long-term thermoluminescent applications [21,22,23,24]. Cerium (Ce) belongs to the lanthanide series and has the electronic configuration 4f^1^ 5d^0^ 6s^2^ in its neutral state. When ionized to Ce^3+^, it loses three electrons, leaving a single electron in the 4f orbital [25]. This 4f^1^ electronic configuration is responsible for Ce^3+^’s unique luminescence properties [26]. The Ce^3+^ ion exhibits transitions between its 4f ground state and the higher-lying 5d excited states. Unlike many other lanthanides, Ce^3+^ has a single 4f electron, leading to broad allowed 4f → 5d transitions, which result in fast luminescence decay times (in the nanosecond range) [27]. The numerous distinct luminescence emission lines observed in Ce^3+^ rare earth ions arise from transitions within the incomplete 4f shell across various low-lying energy levels. The 4f electrons of the Ce^3+^ dopant are effectively shielded from the external environment by the 5s^2^ and 5p^6^ electron shells. Consequently, the 4f states remain unaffected by the LiF host, resulting in emission spectra that remain largely constant in host lattices surrounding SiO_5_. Since the transitions are predominantly radiative, Ce^3+^-doped materials are increasingly favored as luminescent phosphors [28,29,30,31]. This study systematically investigates the TL properties of LiF:SiO_5_:Ce^3+^ phosphors, focusing on their structural and electronic characteristics after beta irradiation. By analyzing the glow curve behavior, dose–response linearity, and heating rate dependence, we aim to comprehensively understand the material’s potential for dosimetry applications. The impact of Ce^3+^ doping on crystal strain, stress, and defect formation is explored using X-ray diffraction (XRD) analysis. The findings contribute to the ongoing development of advanced phosphor materials with superior radiation sensitivity and thermal stability.

## 2. Results and Discussion

### 2.1. X-Ray Diffraction (XRD) Pattern Studies

**Figure 1:**
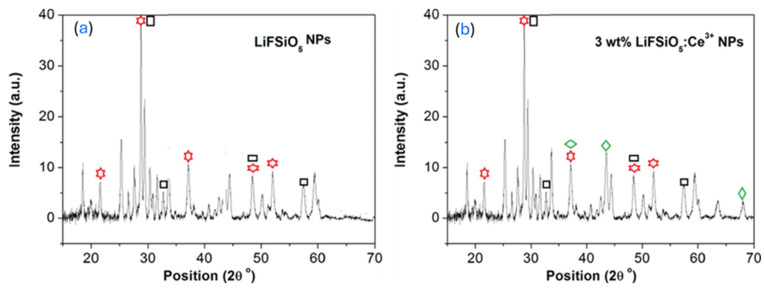
X-ray diffraction patterns of the solution process-based synthesis of (**a**) LiF:SiO_5_ and (**b**) LiF:SiO_5_ doped with Ce^3+^ phosphors (red: LiF; black: SiO_5_, and green: Ce^3+^).

Figure 1 shows X-ray diffraction (XRD) patterns for (a) LiFSiO_5_ nanoparticles (NPs) and (b) LiFSiO_5_:Ce^3+^ (3 wt%) NPs. The overall peak positions remain similar, implying that the main crystalline structure of LiFSiO_5_ is preserved upon Ce^3+^ doping. However, in (b), additional peaks (green diamonds) suggest the possible formation of new minor phases due to Ce^3+^ incorporation, which may indicate secondary phase formation or lattice distortions caused by dopant incorporation. The morphological and optical characteristics of powder phosphor doped with Ce^3+^ were used to characterize the LiF:SiO_5_ material. Figure 1b depicts the measured XRD pattern for angles 2θ ranging from 5° to 70°. The nine prominent di reaction peaks are identified in JCPDS cards 04-0850 and 34-0529 at 2θ values of 21.5°, 28.78°, 33.66°, 37.14°, 43.52°, 48.47°, 52.04°, 57.46°, and 68.05°, respectively. These diffraction peaks represent the beam’s re-reactions from the (211), (401;111), (200), (111;411), (200), (513;331), (321), (422), and (220) planes of the cubic phase of LiF:SiO_5_ doped with Ce^3+^ nanoparticles. Using the Scherrer equation [32], the crystalline size (D) was calculated from the XRD diffraction peaks. The crystalline size of the nanoparticles created in the LiF:SiO_5_ doped with Ce^3+^ phosphor were calculated using the full width at half maximum (FWHM) of the prominent peaks using Equation (1) [33]:

(1) $$D=\frac{k\lambda }{\beta cos\theta }$$

(2) $$d_{hkl}=\frac{n\lambda }{2sin\theta }$$

where k is the crystal width constant at (0.94), (hkl) is the Miller indices from the JCPDS reference files, θ is the Bragg angle, λ is the X-ray wavelength, β is the full width at half-maximum (FWHM), and n is an integer. Scherrer’s formulas were used to calculate the mean crystallite sizes and d spacing.

FWHM is expressed in radians, and θ represents the Bragg’s direction angle in degrees, and all constant values of the shape factor are represented by k. The average crystalline size was calculated from the X-ray line broadening using the Scherrer equation and a Rich Seifert diffractometer with CuKα (λ = 1.5418). Additionally, the inter-planar spacings (d-spacing, dhkl) were extracted from the XRD data using Bragg’s Law [32,34], where n is the direction order of LiF:SiO_5_ doped with Ce^3+^ nanoparticles. The results of the first order XRD diffraction pattern of the SiO_5_ doped with Ce^3+^ NPs inter-planar spacings are between 1.38 and 4.12, which were calculated using the X-ray measurement and are presented in Table 1. From the estimated crystalline size (D), the dislocation density (δ), microstrain (ε), and stress (σ) were calculated.

(3) $$\delta =\frac{1}{D^{2}}$$

(4) $$\varepsilon =\frac{\beta }{4tan\theta }$$

(5) δ=Cε

It is possible to calculate stress from the reported macrostrain using Hooke’s law, where C = 1.46 × 10^10^ Nm^−2^ is the bulk Young’s modulus [35]. Many kinds of laws can occur, and it is obvious that the LiF’s natural growth or manufacturing process plays a role. The type of imperfection that dominates the material as-synthesized will be significantly influenced by SiO_5_ doped with Ce^3+^ crystal. Line deform-like dislocations, such as complex deformities representing boundaries between slipped and unclipped lattice planes, may also allow for energy levels within the forbidden gap. The valence and conduction bands extend throughout the crystal, whereas the defects states are centered on the defects themselves and are referred to as “localized energy levels”. The width of the forbidden gap may also change due to the local dilation and compression zones, resulting in a change in the lattice constant of LiF. In some instances, such as when there is severe distortion around dislocations, large clusters of line deformities, or when impurities precipitate in a second phase, SiO_5_ doped with Ce^3+^ phosphor may be affected [36].

**Figure 2:**
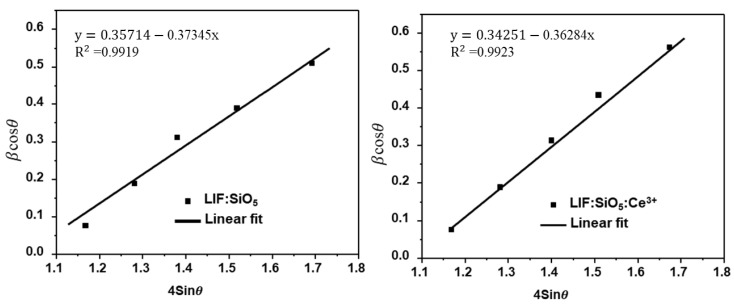
Williamson -Hall (W–H) plots for LiF:SiO_5_ and Ce^3+^-doped LiF:SiO_5_.

The flaws in the lattice structure are responsible for the formation of electron traps. A typical defect, specifically a negative ion vacancy that serves as an electron trap, can be generated by doping SiO_5_ with the ion Ce^3+^ within a LiF phosphor matrix. Thermal vibrations of the lattice ultimately facilitate the escape of trapped electrons, as described by the Maxwell–Boltzmann distribution. However, these vibrations intensify with increasing temperature, leading to a rapid increase in the likelihood of electron eviction. Consequently, trapped electrons are released quickly within a narrow temperature range. This process gives rise to a phenomenon known as thermoluminescence (TL), which occurs when some of the released electrons undergo radiative recombination with the “holes” that have been trapped. The crystalline size of pure and Ce^3+^-doped LiF:SiO_5_ was determined using the Scherrer equation and compared with the Williamson–Hall (W-H) method (Figure 2).

For Ce^3+^-doped LiF:SiO_5_, the average crystalline size was 5.81 nm using the Scherrer equation and 5.97 nm using the W–H method. However, the average crystalline size of un-doped Ce^3+^ was found to be 6.54 nm and 6.65 nm when calculated using the Scherrer equation and the Williamson–Hall (W–H) method, respectively. The results from both methods are in close agreement, differing only slightly at the decimal level. The reduction in crystalline size upon Ce^3+^ doping suggests that the incorporation of Ce^3+^ ions disrupts the normal crystal growth of LiF:SiO_5_. This occurs as foreign Ce^3+^ atoms integrate into the LiF:SiO_5_ lattice, leading to structural modifications.

The Williamson–Hall (W–H) formula is applied as follows [36]:

(6) $$\beta cos\theta =\frac{K\lambda }{D}+4\varepsilon sin\theta$$

The intercept and slope of the plot indicate the kλ/D and lattice strain, respectively. In addition, k, λ, and ε, and represent the shape factor, which is 0.94.

### 2.2. Trap-Emptying Process Dynamics

**Figure 3:**
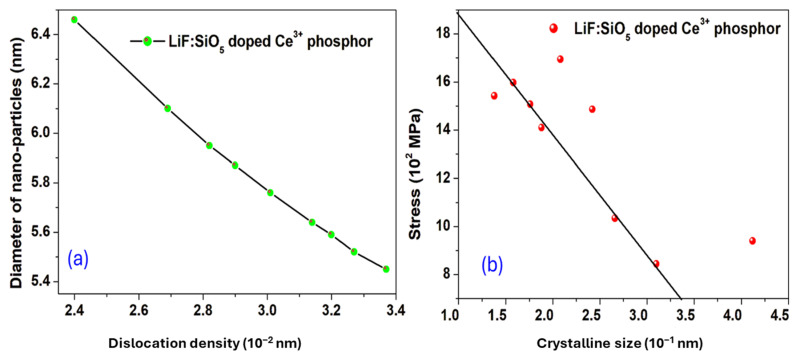
The relationship between (**a**) crystalline size and dislocation density, and (**b**) inter-planar spacing and stress of LiF:SiO_5_ doped with Ce^3+^ solid powder crystal.

Figure 3a depicts the correlation between crystalline size and dislocation density, while Figure 3b illustrates the relationship between crystalline size and stress. The liberation of charge carriers, specifically the release of trapped electrons or holes, represents the most critical step in the thermoluminescence (TL) emission process. Charge carriers can be released through one of two mechanisms, both influenced by the thermal stimulation process, which necessitates multiple energy transfers. The force required to liberate charge carriers is defined as the thermal activation energy. Observations indicate that the optical activation energy consistently exceeds the thermal activation energy. The configuration coordinates of the traps transition from their baseline state to an excited state, resulting in a corresponding average thermal energy:

(7) $$E=\frac{3}{2}K_{B}T$$

The fraction of particles with thermal energy of 1 eV above the ground level is expressed as follows:

(8) $$N=N_{o}Exp\left(\frac{-E}{KK_{B}T}\right)$$

The average thermal energy available at 64 °C and 134 °C is only 0.0435 eV and 0.0526 eV, respectively, which is much less than 1 eV (the activation energy) provided by the Maxwell-Boltzmann distribution for a system in equilibrium at temperature *T*.

This indicates that for E = 1 eV, this fraction would be approximately 10^−15^ and 10^−13^, respectively, at temperatures of 64 °C and 134 °C for the LiF:SiO_5_ doped with Ce^3+^ sample. As reported by FG Major et al. [37], only a small percentage of the population of trapped charges can escape the trap. In this study, the activation energy of LiF:SiO_5_-doped Ce^3+^ was determined to be 0.284 eV, notably higher than the activation energies reported for other Ce^3+^-doped materials. The activation energy of 0.1938 eV observed for Y_2_SiO_5_:Ce^3+^ thin films [38] indicates relatively lower thermal stability, implying that charge carriers in this material can escape more easily than in our LiF:SiO_5_:Ce^3+^ system. Similarly, for Y_3_Al_5_O_12_:Ce^3+^, an activation energy of 0.2401 eV was reported [38], which is still lower than that of our material. This suggests that LiF:SiO_5_:Ce^3+^ has superior thermal stability, likely attributed to the structural effects of SiO_3_^+^ doping within the LiF matrix [39]. These findings demonstrate that the higher activation energy of LiF:SiO_5_:Ce^3+^ may provide enhanced thermal stability, which could be advantageous for applications requiring long-term durability and resistance to thermal degradation. However, this amount of Maxwell–Boltzmann (MB) energy is so insignificant that it has no effect on the traps’ total population of charge carriers. Nonetheless, the traps empty in “no time”, as the name implies, when the sample temperature of LiF:SiO_5_ doped with Ce^3+^ reaches the peak temperature of the glow curve shown in Figure 3a. A factor responsible for this is that a handful of energetic electrons (E ≥ 1 eV) attempt to enter the conduction band exceptionally quickly. Figure 3b shows that stress values range from 8.44 MPa to 16.94 MPa, showing an increasing trend with decreasing crystallite size. This trend suggests that smaller crystallites experience higher internal stress, which could influence mechanical stability and optical properties.

### 2.3. TL Luminescence Characteristics Studies

The primary goal of measuring and analyzing the TL light curves is frequently the extraction of numerous parameters that can be used to define the TL process in the LiF:SiO_5_ doped with Ce^3+^-based phosphor material. For the TL traps, these three parameters, namely, the activation energy (E), the order of kinetics (μ), and the frequency factor (s), are particularly significant.

#### Simple Model for Thermoluminescence

**Figure 4:**
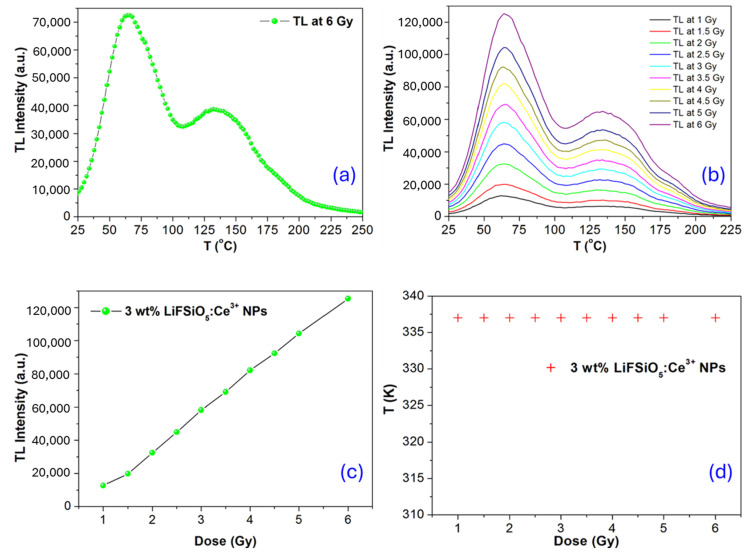
(**a**) TL intensity as a function of temperature is plotted as a glow curve at 6 Gy. (**b**) TL glow curves for different doses from (1–6 Gy). (**c**) TL Intensity vs. at different doses (3 wt% of Ce^3+^-doped on LiFSiO_5_ NPs). (**d**) Peak temperature vs. different doses.

Until significant single crystals of CdS emerged in the late 1940s, no experimental evaluations of the hypothesis relationships between photoconductivity and luminescence were possible [40]. They were the first powder-only synthetic phosphors of the sulfide type. However, the energy band model’s early theoretical treatments were used to explain luminescence. In point of fact, it was so well received that a quantitative theory of the kinetics of phosphorescence and thermoluminescence could be developed [41,42,43]. The band gap energy is responsible for the ionization of valence electrons because matter absorbs the radiation’s energy at a rate of cap Eg is greater than the rate of Eg−Ev (i.e., greater). This delivers free openings and electrons in the conduction band and valence band. With or without their respective delocalized bands, the free carriers could combine, be trapped, or remain free. The phenomenon that lies at the heart of the TSL process is typically explained by looking at the band structure of the electronic transition in an insulating material that exhibits TL luminescence [44]. Figure 4 depicts the process that can occur in the LiF:SiO_5_ doped with Ce^3+^ phosphor both during and after exposure to ionizing radiation. This process is easiest to visualize. To detect TL temperatures, such as 300 °C, we observe how the luminescence changes with temperature when a sample is heated to a constant temperature. The so-called” glow curve” of the TL signal indicates the presence of electron traps in the sample by showing distinct peaks at various temperatures.

As seen in Figure 4a, the TL glow curve at 6 Gy represents the thermoluminescence (TL) intensity as a function of temperature (T) for a radiation dose of 6 Gy. The TL intensity increases with temperature, reaches a maximum at around 75 °C, and then decreases. A second peak is observed at a higher temperature (around 140–150 °C), suggesting the presence of multiple trap levels in the material. The glow curve shape and peak positions indicate the energy levels of electron traps in the material. The first peak corresponds to a shallower trap (lower activation energy), while the second peak indicates deeper traps. Figure 4b shows TL glow curves for different radiation doses, ranging from 1 Gy to 6 Gy. The TL intensity increases as the dose increases, showing that more charge carriers are trapped and released upon heating. The overall shape of the curves remains similar, but the intensity scales proportionally with dose. The primary peak (~75 °C) remains at the same temperature for all doses. This indicates that the material follows first-order kinetics, meaning that the trapping and recombination process remains consistent across different doses. This graph is crucial for dose calibration in radiation dosimetry applications. A linear relationship is observed in Figure 4c, which confirms that LiFSiO_5_:Ce^3+^ NPs can be used as a dosimetric material, as it provides a reliable method to measure radiation exposure. The peak temperature remains constant (~335 K or ~62 °C) in Figure 4d, meaning that the trap depth remains unchanged with increasing dose. This stability in peak position is desirable for TL materials, as it ensures reproducibility and reliability in radiation dose measurements.

No re-trapping exists because all electrons released from traps undergo TL transitions, as stated by Randall and Wilkins [45]. A “first-order” reaction is a reaction in which the rate of release is thought to be inversely proportional to the concentration of trapped charge. Consider a material with defects that produce a single electron trap level at temperature T (in kelvin) and time t, where E is the activation energy and n electrons represent the trap depth. Given the Maxwell–Boltzmann distribution’s description of the energy distribution of electrons within the trap, the probability that an electron will be released from the trap is given by the Arrhenius equation. The probability of escaping the traps is given as follows:

(9) $$p=sExp\left(\frac{-E}{kT}\right)$$

where the frequency factor is s. As a result, the fraction $\frac{N}{N_{o}}$ equals 10^−13^ and 10^−15^, or s. Only the insignificant fractions 10^−13^ and 10^−15^ can escape at any given time while the glow peak is being emitted. As a result, s is an attempt to escape factor or frequency factor, and k is the Boltzmann constant value between 10^13^ and 10^15^ s^−1^, which is the lattice vibration frequency of LiF:SiO_5_ doped with Ce^3+^ crystal. The rate at which the trap’s electrons are released is expressed as follows:

(10) $$-\left(\frac{dn}{dt}\right)=nsExp\left(\frac{-E}{kT}\right)$$

The TL glow’s intensity, *I*(*t*), is influenced by the speed at which electrons are released from traps and transported to luminescence centers.

(11) $$I(t)=-C\left(\frac{dn}{dt}\right)=CnsExp\left(\frac{-E}{kT}\right)$$

Here, C is a luminescence efficiency constant. If we consider the rate of heating, the following is obtained:

(12) $$\beta =\frac{dT}{dt}$$

Equation (11) is now expressed as follows:

(13) $$\frac{dn}{dT}=-\left(\frac{1}{\beta }\right)nsExp\left(\frac{-E}{kT}\right)$$

We obtain the following after integration:

(14) $$ln\left(\frac{n}{n_{o}}\right)=-\int \left(\frac{1}{\beta }\right)sExp\left(\frac{-E}{kT}\right)dT$$

where $n_{o}$ represents the quantity of electrons in the trap at temperature T and time t0, respectively. Lastly, changing n in Equation (12) yields the following:

(15) $$I(T)=-n_{o}sExp\left(\frac{-E}{kT}\right)Exp\left(\frac{-s}{\beta }\int \left(\frac{1}{\beta }\right)sExp\left(\frac{-E}{kT}\right)dT\right)$$

This is how the glow intensity I of an electron trapped at a single trapping level, E, is expressed. It is a Randall and Wilkins (RW) expression for first-order (monomolecular) kinetics, and the glow curve has an asymmetric shape, being wider on the low-temperature side than on the high-temperature side. When the derivative of Equation (16) is set to zero (i.e., ${\left(\frac{dI}{dT}\right)}_{T}=T_{m}=0$), the following is obtained:

(16) $$\frac{\beta E}{kT_{m}^{2}}=sExp\left(\frac{-E}{kT}\right)$$

where Tm is the temperature at which the glow peaks. The thermal stability of the trapped electrons increases with increasing E and s values, raising the glow peak temperature, according to Equations (10) and (17).

The general order kinetics (GOK) model describes a more general form of trap kinetics, where the order of the reaction (denoted by *m*) can vary between 1 (first order) and 2 (second order), allowing for the fitting of glow curves with more flexibility. The equation for the GOK model is provided below:

(17) $$\frac{dN}{dt}=A.N^{m}.e^{\frac{-E}{kT}}$$

Here, $\frac{dN}{dt}$ is the rate of change of the number of trapped carriers, N is the number of trapped carriers, *A* is the pre-exponential factor (attempt-to-escape frequency), *m* is the order of the kinetics (where *m* = 1 for first-order and *m* = 2 for second-order kinetics), *E* is the activation energy of the trap, *k* is the Boltzmann constant, and *T* is the temperature. The GOK model is more flexible as it can describe both first- and second-order behavior as well as intermediate cases, depending on the value of *m*.

Equation (18) assumes that the overall glow curve can be represented as a sum of individual components, each corresponding to a specific trap or set of traps with a characteristic activation energy.

The general form of the glow curve deconvolution (GCD) equation is expressed as follows:

(18) $$I(T)=\sum_{i=1}^{n}A_{i}.\left(\frac{T}{T_{m,i}}\right).exp\left(-\frac{E_{i}}{KT}\right).exp\left(-\frac{T}{T_{m,i}}\right)$$

I(T) is the intensity of emitted light (thermoluminescence signal) as a function of temperature T, A_i_ is the pre-exponential factor (intensity coefficient) for the *i* the glow peak, T_m,I_ is the temperature corresponding to the maximum of *i* glow peak (often determined from the glow curve), *E*_i_ is the activation energy associated with the *i*^th^ trapping center, α is a constant that depends on the distribution of the trap depths, k is the Boltzmann constant, and T is the temperature at which the intensity is measured.

The value of E found using the Maxwell–Boltzmann distribution (MB) method is unaffected by the dynamics of the TL glow peak. The presence of thermal quenching affects the value of E produced with the MB method compared to the RW method. The RW technique works best with samples that have received minimal doses of radiation or are not close to saturation conditions. The method offers a possible strategy for adjusting the value of E. The test dose used to create the TL light curve is our “probe” of the LiF:SiO_5_ doped with Ce^3+^ phosphor material. The best fit is shown in Figure 4b,d, which has the highest linear regression value (R2).

The thermoluminescence (TL) properties of lithium fluoride (LiF)-based phosphors have been extensively studied, with various dopants enhancing their dosimetric performance (Table 2). Table 2 lists different compositions of LiF phosphors with dopants like Mg (magnesium), Cu (copper), P (phosphorus), B (boron), Si (silicon), and Ce (cerium). These dopants influence the TL response of the phosphors. Higher TL peak temperatures suggest better thermal stability, while lower temperatures indicate suitability for detecting lower-energy radiation. LiF:SiO_5_,Ce^3+^ has a significantly lower TL peak in the range of 140–150 °C, which suggests it has shallower traps and could be more suitable for low-temperature TL applications.

**Figure 5:**
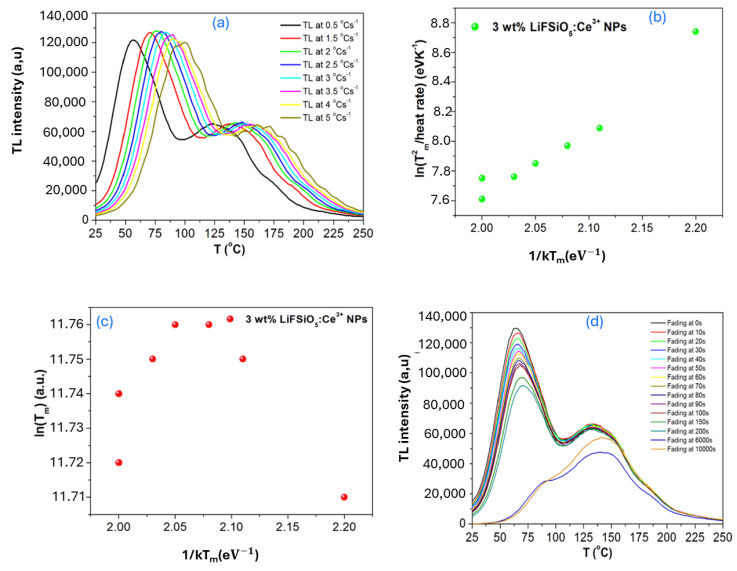
(**a**) Effect of the heating rate beta radiation on the shape and the position of a first-order TL glow curve, (**b**) ln ($T_{m}^{2}$/heating rate) vs. 1/kTm, (**c**) ln (T_m_) vs. 1/kT_m_, (**d**) effect of fading on the TL glow curve.

Figure 5 consists of four subplots (a, b, c, and d) that illustrate the effect of the heating rate and fading on the thermoluminescence (TL) glow curve of a material (3 wt% LiFSiO:Ce^3+^ NPs). The subplot in Figure 5a illustrates the impact of different heating rates on the shape and position of a thermoluminescence (TL) glow curve. The TL intensity is plotted as a function of temperature (T) for various heating rates, ranging from 0.5 °C/s to 5 °C/s. The radiation dose is provided in Gy, and the duration is reported (1–6 Gy for 30 min). As the heating rate increases, the glow peaks shift to higher temperatures. This is due to thermal lag, where a higher heating rate requires more thermal energy for the trapped charge carriers to be released. The graph in Figure 5b is used to determine the activation energy (E) of the traps using Equation (15). We conclude from the data that kinetics of μ = 0.519 and 0.563 describe the given TL glow curve (seen in Table 3). We can say that a good approximation of superlinearity can consider this as a first-order kinetic TL peak due to the experimental uncertainties in the data resulting from the lack of data points on the TL glow curve as illustrated in Figure 5b,c. The subplot in Figure 5d investigates how TL intensity changes over time due to fading, which refers to the loss of stored charge carriers from traps at room temperature. As time progresses (from 0 s to 10,000 s), the TL intensity decreases, indicating that some charge carriers escape the traps without contributing to the luminescence. Faster fading at lower temperatures suggests shallow trap levels that allow thermally stimulated de-trapping even at room temperature. Two peaks indicate that some trap levels are more stable than others. The high-temperature peak is less affected by fading, corresponding to deeper traps.

Charge carriers can spontaneously fade out of traps at room temperature as depicted in Figure 5d.

(19) $$I=I_{o}e^{-pt}$$

The peak region at time t is denoted by the symbol *I*, and the peak at time t=0 is denoted by $I_{o}$ when the first-order expression is viewed as a function of time. The fading factor p is provided as follows:

(20) $$p=-\frac{1}{t}ln(\frac{I}{I_{o}})$$

**Figure 6:**
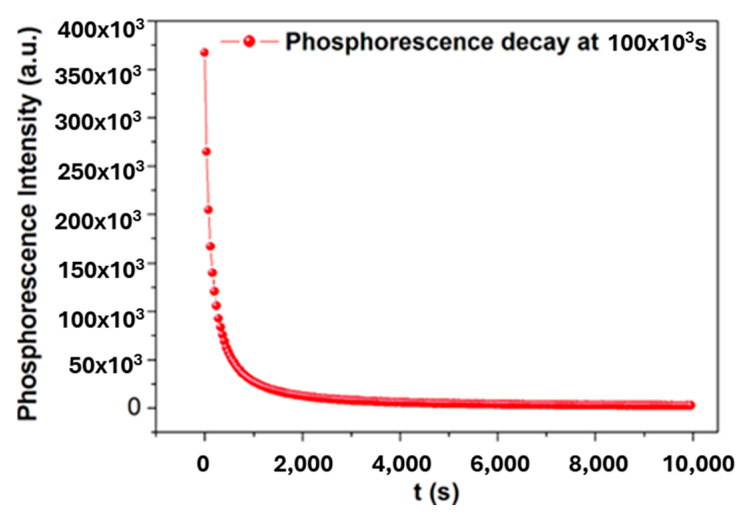
Phosphorescence of LiF:SiO_5_ doped with Ce^3+^ nanoparticles.

Fluorescence is a luminescent process that lasts only as long as the excitation is maintained for LiF:SiO_5_ doped with Ce^3+^ phosphor. Therefore, temperature does not affect the speed at which fluorescence decays. A luminescent phenomenon known as phosphorescence can be observed after the exciting source of beta radiation is removed. Temperature impacts brightness because some levels of E may entrap it in the prohibited gap. A rise in temperature causes an increase in the amount of energy required for trapping. Figure 6 depicts the phenomenon of phosphorescence. The LiF:SiO_5_ doped with Ce^3+^ phosphor material can be seen in action in the unique solid qualities acting according to the heating rate. However, the glow peak shifts to higher temperatures due to the thermal quenching effect, whose effectiveness increases with temperature (seen in Figure 6). Typically, the efficiency of luminescence is a temperature-dependent variable, which indicates that efficiency decreases with increasing temperature. The opposition between radiative advances, which are almost temperature autonomous, and non-radiative changes, which get more grounded as it gets more sweltering, is the way the heat extinguishing impact is explained.

## 3. Experimental Section

### 3.1. Preparation of Materials and Methods

The following materials were used to synthesize LiF:SiO_5_:Ce^3+^ phosphor: lithium fluoride (LiF) (Sigma Aldrich, Wuxi, China, 98.5%), silicon dioxide (SiO_2_) (MSE Supplies, Tucson, AZ, USA, 99.99%), and cerium nitrate hexahydrate (Ce(NO_3_)_3_·6H_2_O) (Noah Chemicals, San Antonio, TX, USA, 99.99%). Ethanol is used as a precursor solution. An alumina crucible is used for heating and sintering. A controlled atmosphere with argon or nitrogen gas flow was used for stabilizing the Ce^3+^ oxidation state. Lithium fluoride (LiF) and silicon dioxide (SiO_2_) were mixed in a 1:1 molar ratio. Ethanol was used as the precursor solution. To optimize TL properties, cerium nitrate hexahydrate (Ce(NO_3_)_3_·6H_2_O) was added to the mixture at 3 mol% Ce^3+^ concentration. Ce(NO_3_)_3_·6H_2_O was first dissolved in ethanol to ensure uniform distribution. The components were thoroughly mixed using a mortar and pestle for 1 h to obtain a homogeneous precursor powder. The mixture was transferred into an alumina crucible and preheated at 40 °C for 2 h to remove volatile impurities and initiate precursor reactions. The pre-calcined powder was further sintered in a furnace at 700 °C for 4 h. The process was conducted under a controlled atmosphere (argon or nitrogen flow) to stabilize the Ce^3+^ oxidation state. The sintered product was ground again to obtain fine LiF:SiO_5_:Ce^3+^ phosphor particles.

### 3.2. Characterization

Using Cu Kα (λ = 1.5418 Å) radiation and a D8 advanced X-ray diffractometer (XRD; Bruker, Germany), the crystallinity of LiF:SiO_5_:Ce^3+^ nanophosphor was deter-mined. The generated current was 30 mA, while the accelerated voltage was 40 kV. Data are gathered every 0.01° in the 15–80° angle range in 2θ. A field emission scanning electron microscope (FE-SEM, JEOL JSM-7800F, Tokyo, Japan) was used to view the particle morphology. A PC-controlled thermoluminescence dosimeter reader with a CCD (charge-coupled device) spectrometer (TL 1009I, NUCLEONIX SYSTEMS, Secunderabad, India) was used to record thermally stimulated luminescence (TSL) glow curves and emission spectra. A UV lamp with a wavelength of 254 nm (Advanced Technology Inc., ATICO, Newport News, VA, USA) was used to irradiate the sample. About 12 cm separates the sample and the source during the irradiation period. The samples were initially heated at a constant rate of 2 °C/s and exposed to radiation for 20 min before the glow curves were measured.

## 4. Conclusions

The accompanying outcomes are gotten in the speculative situation of awesome heat contact between the LiF:SiO_5_ doped with Ce^3+^ test thermoluminescent and the heating component: the greatest temperature at the pinnacle, Tm, shifts to higher temperature values with increasing heating rate. As the heating rate expands, the thermoluminescence force, top region, and pinnacle level diminish. Moreover, heating the sample lets electrons out of its electron traps. A portion of these electrons can then relocate to iridescent focuses, and when they do, light (i.e., TL) is delivered because of recombination into these focuses from LiF:SiO_5_ doped with Ce^3+^ phosphorus. (i) Ionization results from openness to radiation from beta-radiation sources. (ii) The capacity of radiation energy over the long run, on the off chance that spillage is negligible, indicates that the lifetime of the electrons in the bands should be significantly longer than the sample’s time spent away. Its energy profundity E affects the band’s lifetime underneath the conduction band. This study focuses on LiF:SiO_5_ doped with Ce^3+^ phosphor yet lacks an in-depth analysis of defect dynamics and long-term trap stability under varying environmental conditions. Future research should explore advanced TL modelling, dopant optimization for enhanced sensitivity, and real-world dosimetric applications, ensuring broader applicability in radiation detection.

## Figures and Tables

**Table 1:** Analysis of XRD microstructural measurements for LiF: SiO_5_ doped with Ce^3+^ nanoparticles.

Peak Pos (2*θ*°)	FWHM (*β*)	d-Spacing	D (nm)	*δ* (10^−2^ nm^−2^)	*ε* (10^−2^)	*σ* (10^2^ MPa)
21.57	0.2622	4.12	5.45	3.37	6.44	9.40
28.78	0.2388	3.10	5.52	3.28	5.78	8.44
33.66	0.2959	2.66	5.59	3.20	7.08	10.34
37.14	0.4294	2.42	5.64	3.14	10.18	14.86
43.52	0.4996	2.08	5.76	3.01	11.60	16.94
48.47	0.4236	1.88	5.87	2.90	9.66	14.10
52.04	0.4592	1.76	5.95	2.82	10.32	15.07
57.46	0.4992	1.58	6.10	2.69	10.94	15.97
68.05	0.5097	1.38	6.46	2.40	10.56	15.42

**Table 2:** The comparison of TL parameters with previous studies on LiF-based phosphors.

Phosphor Composition	Main TL Peak Temperature (°C)	Linear Dose Response Range	Ref.
LiF:Mg, Cu, P, B	220	Up to 10 Gy	[46]
LiF:Mg, Cu, P, Si	220	Up to 10 Gy	[46,47]
LiF:Mg, Cu, P	205	Not specified	[47]
LiF:Mg, Cu	205	Not specified	[46]
LiF:Mg, Cu, P	202	Low dose range	[47]
LiF:SiO_5_, Ce^3+^	140–150	Up to 6 Gy	Present

**Table 3:** The LiF:SiO_5_ doped with Ce^3+^ nano-crystalline phosphor exposed to beta radiation has kinetic and trapping characteristics.

Temperature (°C)	τ (K)	δ (K)	ω (K)	μ	Exp $\left(\frac{-E}{KT}\right)$	s (s^−1^)
64	39	42	81	0.519	1.096 × 10^−15^	1.096 × 10^−15^
164	28	36	64	0.563	4.100 × 10^−13^	1.096 × 10^−13^

## Data Availability

Data are contained within the article.
